# Neural correlates of transient topographical disorientation: an experimental EEG–MRI case study

**DOI:** 10.1007/s00415-023-11902-z

**Published:** 2023-08-11

**Authors:** Paul Theo Zebhauser, Marine Vernet, Sandra Nischwitz, Philipp G. Sämann, Anna-Katharine Brem

**Affiliations:** 1 Department of Neurology, Klinikum Rechts der Isar der Technischen Universität, Munich, Germany; 2 Max Planck Institute of Psychiatry, Munich, Germany; 3 IMPACT Team, Lyon Neuroscience Research Centre (CRNL), INSERM U1028, CNRS UMR5292, Université Claude Bernard Lyon 1, Lyon, France; 4 Department of Old Age Psychiatry, Institute of Psychiatry, Psychology and Neuroscience, King’s College London, London, SE5 8AF UK; 5 University Hospital of Old Age Psychiatry, University of Bern, 3008 Bern, Switzerland

## Introduction

Topographical disorientation (TD) is a cognitive dysfunction characterized by impaired wayfinding within large-scale surroundings and has been described in the context of various neurological conditions [1–3].

Topographical orientation is generally linked to a network evolving around the right parahippocampal gyrus and medial temporal lobe, fitting well with reports of isolated TD after lesions to these hubs [4, 5]. Besides lasting manifestations, reports of transient TD (TTD) have been published. This condition is usually characterized by short-lasting episodes of TD, occurring only once to a few times, which could occasionally be traced back to ischemic events [6] or migraine aura [7]. In some patients, epilepsy was suspected, but routine clinical EEG did not show any conclusive evidence [8].

Here, we present a case of TTD with a previously unreported high frequency of events. We assumed epilepsy as the underlying etiology and hypothesized that underlying epileptic activity could be detected more reliably by task-related compared to resting state EEG (rsEEG). We, therefore, recorded EEG during real and imaginary wayfinding-tasks.

### Case

A 47-year-old otherwise healthy male presented to our clinic with frequent (around 20) daily episodes of TD, lasting for up to 20 s. No aura-like symptoms were present, although frequency of episodes increased when being under stress or sick. He described the episodes as follows:

*It usually happens when I am in the car. For example, when I am driving home from work, I suddenly do not know which way to go. It then takes some time for me to reorient. Strangely, I recognize the surroundings, but do not know the way!*

He reported worries and insecurities regarding these events and described limitations in everyday activities, such as ceasing to pick up friends in the car.

Neurological/psychiatric evaluation was normal, comprehensive neuropsychological testing revealed average-to-superior results. Particularly, visuo-spatial-functioning was unimpaired (*z* scores − 0.7 to + 2.1 for mental rotation, block-tapping, route learning).

**Fig. 1:**
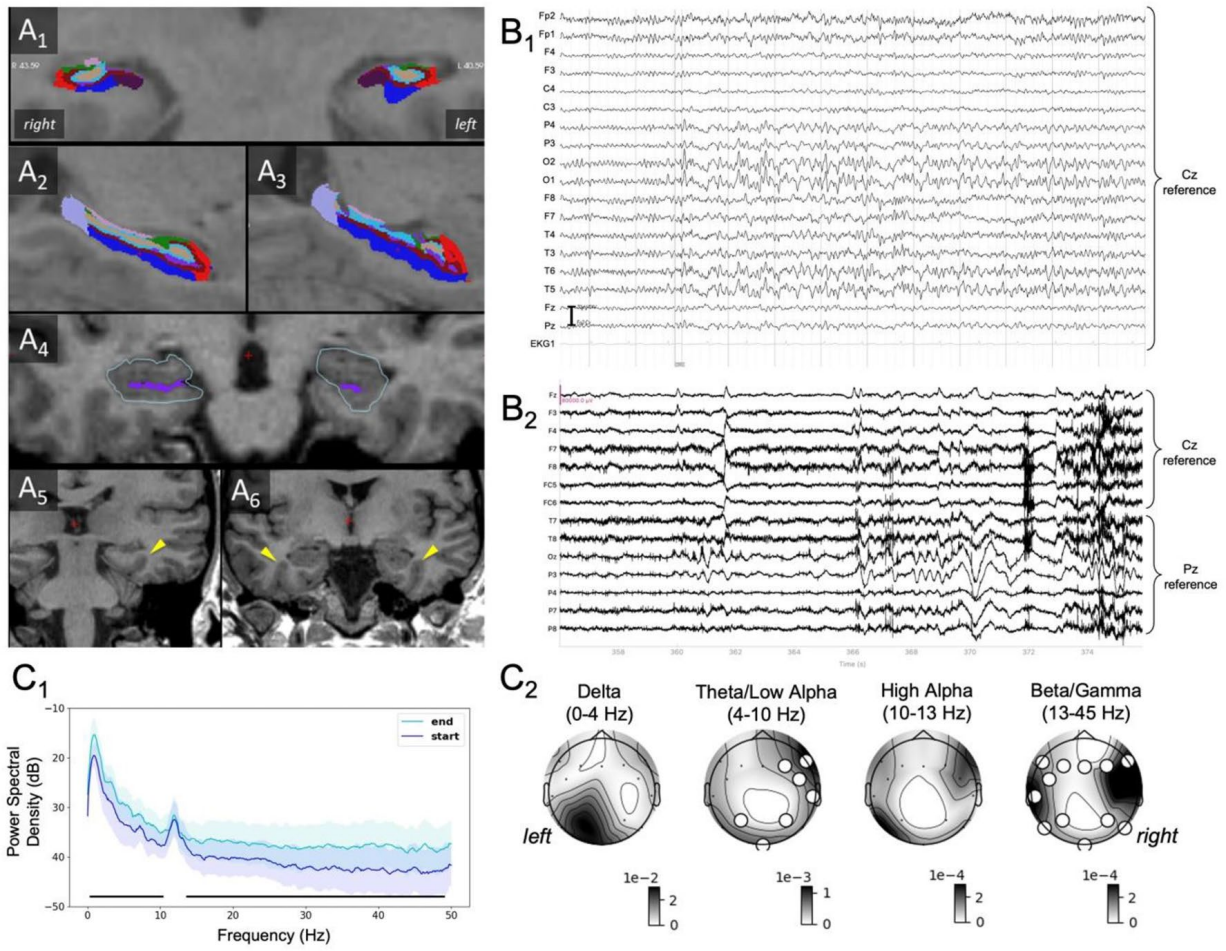
Structural MRI showing a partial, incomplete hippocampal inversion. **A1** Coronal view of hippocampal subfields (radiological view). Note asymmetry of subiculum. **A2** Sagittal view of right hippocampal subfields. **A3** Sagittal view of left hippocampal subfields. Note thickened and irregularly shaped left subiculum. **A4** Note more roundish, pyramidal shape of left hippocampal contours and shorter left hippocampal fissure, pointing out different internal hippocampal structure. **A5** More verticalized and deeper left occipitotemporal sulcus in the posterior hippocampus (yellow arrow). **A6** Same as (A5) in the more anterior hippocampus, yet bilaterally. B Clinical and experimental EEG. **B1** Clinical resting state EEG: normal alpha-background activity is disrupted by a right occipitotemporal sharp wave (O2 > O1, T6, P4), followed by bilateral delta/theta activity for 7 s. EEG signal is referenced to Cz. No clinical symptom is associated with such activity because the patient was not performing any task. **B2** Task-related EEG, samples of the last 20 s of a recording with TD, containing epileptiform activity according to both evaluators (from Exp 1). The EEG signal was re-referenced to Cz (frontal electrodes) or Pz (temporo-parieto-occipital electrodes). **C** Power spectra and topographical maps for frequency bands. **C1** Power Spectrum Density in dB (average and standard deviation across all electrodes) for the start and end of recordings with TD. Black lines with stars below the plots indicate the frequencies for which the cluster permutation test revealed a statistically significant difference between the two compared conditions. **C2** Power topographical maps of recordings with TD in different frequency bands (note that the high alpha band corresponds to the alpha peak of this patient). *R/L* right and left (neurological convention). The maps show the power difference between the end and the start of the recordings. The position of the electrodes is indicated with a black dot. Larger white dots indicate electrodes belonging to a cluster showing a statistically significant difference between the start and the end of the recordings. Note the apparent lateralization of slow activity in the right temporo-occipital region associated with TD episodes

Assessment of standard MRI sequences supported by hippocampal subfield segmentation (FreeSurfer v7.1.1; http://surfer.nmr.mgh.harvard.edu) revealed minor left hippocampal abnormalities, interpretable as partial incomplete hippocampal inversion (IHI, Fig. 1A1) characterized by (1) a more rounded shape of the contours of the left anterior hippocampus compared with the right side (Fig. 1A4), (2) a verticalized deep occipitotemporal sulcus particularly on the left side and more clearly in the anterior section (Fig. 1A5–6), (3) an abnormally shaped and thickened left subiculum (Fig. 1A2–3 [blue subregion]), and (4) a shallow left hippocampal fissure (purple line, Fig. 1A4) not opening to the choroidal fissure. No hippocampal sclerosis and no other brain abnormality was detected.

2 × 20 min rsEEG (unmedicated) revealed two episodes of bilateral occipitotemporal delta/ theta activity preceded by bilateral sharp transients (Fig. 1B1). At the beginning of the episodes, abnormal activity was most prominent in electrode O2, suggesting a possible right-hemispheric epileptogenic focus, before becoming bilateral.

Based on the paroxysmal clinical presentation and experimental EEG findings (see below), the patient was started on levetiracetam (1500 mg/day) which was discontinued by the patient before follow-up due to lack of efficacy. While clinical presentation, MRI and EEG overall suggested an epileptic origin of complaints. We, therefore, employed task-related EEG to identify neurophysiological correlates of TD-episodes and evaluate the potential lateralization of such activity.

## Task-related EEG

Eyes-open EEG was recorded during real and imaginary wayfinding-experiments. Whenever experiencing TD, the patient was instructed to wait until the episode had passed and indicate nonverbally once he felt reoriented. These episodes were marked in all EEG recordings for further qualitative and quantitative analysis.

### Experiment 1

We first walked the patient around the clinic to ensure agreement on 20 landmarks (e.g., cafeteria, reception). Then, he was placed in a wheelchair and asked to indicate the routes to specific landmarks. At each junction, he signaled the direction using a keypad controlled by his right index finger.

### Experiment 2

We reenacted situations of TD in the patient’s everyday life. He was asked to imagine driving around his hometown, using routes between familiar localities. Prior to this, we prepared a list of landmarks together with the patient (e.g., bakery, his house). His task was to imagine driving from one place to another.

### Experiment 3

The patient was asked to imagine walking around the hospital to find routes along the landmarks from Experiment 1.

### Analysis

EEG was obtained using 16 electrodes (Fz/3/4/7/8, Cz, Fc5/6, T7/8, Pz/3/4/7/8, Oz) and evaluated independently by MV and SN. For quantitative analysis, we used MATLAB with EEGLAB [9] and MNE-Python [10]. The first (last) two seconds of recordings were discarded and the adjacent first (last) 10 s were analyzed (reported TD-episodes lasted between 5 and 15 s in our experiments and recordings were stopped after reorientation). Two-by-two comparisons were made for section (start/end) * type of recording (with/without TD) in the frequency and spatial domain (power spectra averaged across electrodes, topographical power maps for frequency bands). Statistical significance was assessed by cluster permutation tests.

### Results

During 15/35 recordings, the patient reported an episode of TD (5/2/8 recordings for experiments 1/2/3), of which 5 were considered to have epileptiform activity at the end by both evaluators (8 by one, 2 by none Fig. 1B2). Of 20/35 recordings without TD (9/6/5 recordings for experiments 1/2/3), 4 were considered to have epileptiform activity by both evaluators (3 by one, 13 by none). Hence, TD was present in 43% of the navigation tasks and epileptiform activity was detected more often in recordings with TD than in recordings without TD (chi-square-test *p* < 0.001).

Quantitative analysis, based on the patient’s subjective reports of TD (and not on evaluators’ ratings), showed significantly higher broadband power at the end of recordings with TD compared to the start of respective recordings, except for the alpha peak (Fig. 1C1). In contrast, no differences in power spectra were observed for start vs. end of recordings without TD. Using topographical analysis, the comparison between start and end of recordings with TD showed increases of theta and low alpha frequency power over right temporo-occipital electrodes and of beta and gamma frequency power over a bilateral and widespread set of electrodes concurrent with reported episodes of TD (Fig. 1C2).

## Discussion

We report the case of a patient with a previously unreported high frequency of episodes of TD, suggestive of epileptic etiology. While during rsEEG only two abnormal EEG-episodes were detected, we found a clear electroclinical correlation in an experimental setting targeting topographical orientation. TD-associated episodes of ictal activity were coupled with increased oscillatory brain activity, pronounced in theta and low alpha frequencies, and centered around right-hemispheric regions. This EEG-focus is consistent with the literature on impaired wayfinding and TD, for which mostly right (para) hippocampal lesions have been found [11].

Reviewing five single MRI criteria of IHI and the global aspect criterion [12], our case could be classified as left-sided partial IHI, whereas on the right side only a deep occipitotemporal sulcus was noted. The prevalence of left-sided partial IHI has been reported as 11.9% in a young, healthy normal population (right-sided: 9.0%) and full IHI in 17.1% on the left and 6.5% on the right side [12]. In clinical samples the prevalence depends critically on selection criteria and was as low as 1.2% for partial IHI in patients without epilepsy [13]. In patients with epilepsy, the average prevalence of IHI is higher (30%), varying between temporal lobe (25%), Rolandic (44%) and cryptogenic epilepsy (57%), with a strong left-hemispheric emphasis [14]. Scientific evidence indicates that the epileptogenic potential of IHI itself is rather low and the susceptibility to develop seizures likely roots in co-associated subtle cortical malformations [15] or sulcal maldevelopment [12]. As in our case, review work indicates that the laterality of seizure onset in the EEG and the laterality of the IHI do not correspond in many cases [15]. We thus convey that the IHI in this case may further support a (formally cryptogenic) type of focal epilepsy.

In summary, we were able to provide, for the first-time, structural and functional neuroimaging evidence for TTD being associated with a form of focal epilepsy. Importantly, detection of EEG-abnormalities was considerably facilitated when analyzing personalized task-related EEG compared to rsEEG. Hence, our findings underscore the clinical value of task-related EEG in patients with transient subjective neurological phenomena. In this context, we encourage the use of complementary quantitative EEG-analysis for diagnostic purposes in clinical care.

## Data Availability

EEG-data are available upon reasonable request from the corresponding author.

## Code availability

Code for EEG-analysis is available at https://github.com/marinevernet/topographical_disorientation.
